# Supplementary material for: Anas barbariae 200K Modulates Cell Stiffness and Oxidative Stress in Microglial Cells In Vitro
Source: Int J Mol Sci. 2025 Feb 9;26(4):1451. doi: 10.3390/ijms26041451 (PMC11855513; doi:10.3390/ijms26041451)
Supplement: Supplementary file 1 [file ijms-26-01451-s001.zip › ijms-3257083-supplementary.pdf]

Supplementary data

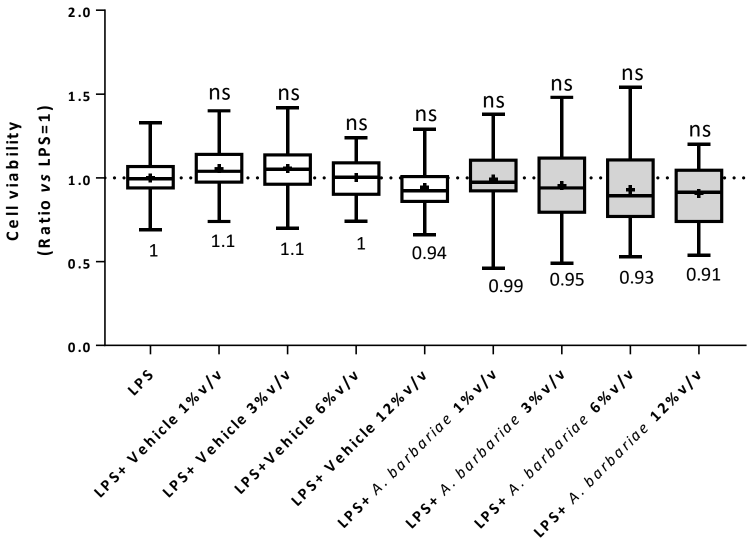

Supplementary Figure S1: Viability of *A. barbariae* 200K and the vehicle in LPS-inflamed primary murine microglial cells

The cell viability was measured using XTT viability assay. The cells were inflamed with 10 ng/mL LPS for 4 h and incubated with *A. barbariae* 200K or the vehicle at 1%,3%,6%, and 12% (v/v) for 20 h. The experiments were repeated three times in an independent manner. Statistical significance: ns = not significant. (LPS: lipopolysaccharide; *A. barbariae*: *Anas barbariae*, vehicle: sterile water).

A

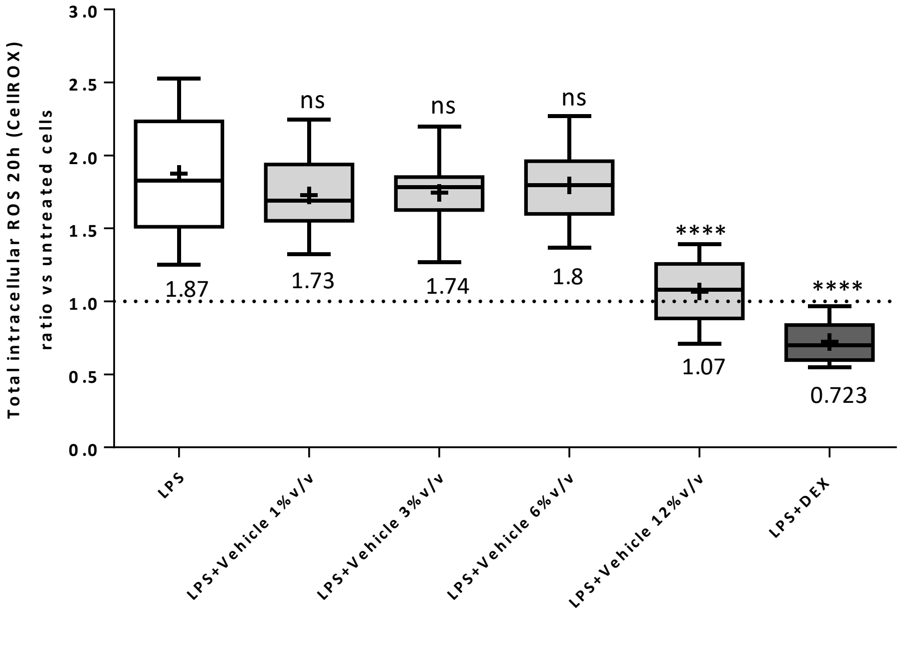

B

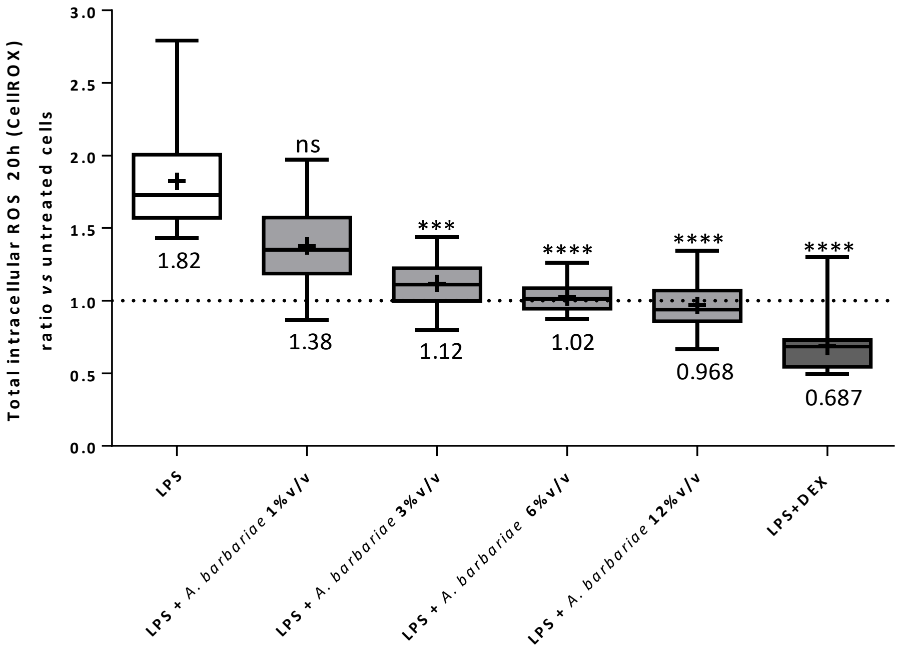

Supplementary Figure S2: Dose-dependent response (A) with the vehicle or (B) with *A. barbariae* 200K on oxidative stress in LPS-inflamed primary murine microglial cells.

The production of ROS was assessed using the CellROX DeepRed probe as described in Materials & Methods. Briefly, the cells were inflamed with 10 ng/mL LPS for 4 h and incubated with or *A. barbariae* 200K (1%,3%,6%, and 12% v/v) for 20 h. (**A**) Total intracellular ROS measured with vehicle. (**B)** Total intracellular ROS with *A. barbariae* 200K. All the values were divided by the fluorescence value of the untreated cells. The dotted line at 1 corresponds to the untreated cells. The experiments were repeated three times in an independent manner. Statistical significance: *** $p < 0.001$, **** $p < 0.0001$; ns = not significant. (ROS: reactive oxygen species; LPS: lipopolysaccharide; DEX: dexamethasone; *A. barbariae*: *Anas barbariae*, vehicle: sterile water)
